# Supplementary material for: Are carbon emissions trading and green financial instruments synergistic? -Comprehensive quantitative research based on content analysis
Source: PLoS One. 2024 Mar 7;19(3):e0298601. doi: 10.1371/journal.pone.0298601 (PMC10919601; doi:10.1371/journal.pone.0298601)
Supplement: S1 Data — (ZIP) [file pone.0298601.s001.zip › Data/地级市Z_F工作报告.zip!/║╙─╧/╨┬╧τ╩╨/└┤╫╘╣1⁄2╓┌║┼í╛┬φ┐╦╩2╛▌═°í┐_2011.docx]

各位代表：

现在，我代表市人民政府向大会作工作报告，请予审议，并请各位政协委员提出意见。

一、“十一五”工作回顾

五年来，市人民政府在省委、省政府和市委坚强领导下，在市人大、市政协监督支持下，团结带领全市人民，深入贯彻科学发展观，锐意进取，扎实工作，开创了经济社会发展的新局面。

过去五年，经济发展持续加快，综合实力迈上新台阶。地区生产总值由544亿元增加到1181亿元，年均增长14.5%，高于全省平均水平1.7个百分点；增速由2005年的全省第12位上升到2010年的第3位。财政一般预算收入由25.4亿元增加到70.5亿元，增加了1.8倍，年均增长22.7%，高于全省平均水平1.9个百分点；总量和增速分别由2005年的全省第7位、第9位上升到2010年的第4位和第3位。地区生产总值和财政一般预算收入总量均提前一年实现翻番目标。规模以上工业增加值由175亿元增加到533亿元，增加了2倍，年均增长22.2%，高于全省平均水平2个百分点；增速由2005年的全省第5位上升到2010年的第1位。全社会固定资产投资4009亿元，是“十五”期间的4.7倍，年均增长32.4%；城镇固定资产投资总量连续8年保持在全省前4位。金融机构存款余额由560亿元增加到1144亿元，年均增长15.4%；贷款余额由322亿元增加到712亿元，年均增长17.2%；存贷款余额均稳居全省第4位。城镇居民人均可支配收入15752元，年均增长13.6%；农民人均纯收入6241元，年均增长14.8%。

过去五年，结构调整力度较大，发展方式明显转变。二、三产业比重较“十五”末提高3.1个百分点；工业化率达到51%，提高8个百分点。落后产能基本淘汰。以壮士断腕的决心，完成10个行业的153户企业200个项目关停任务，基本淘汰小水泥、小造纸、小火电、小化工等落后产能，圆满完成“十一五”节能减排目标任务。产业升级步伐加快。既立足当前，又着眼长远，大力发展“一谷五基地”战略支撑产业和新兴产业，努力抢占新一轮发展的制高点。六大战略支撑产业实现主营业务收入、利润分别占全市工业的61%和55.6%。规模以上工业主营业务收入突破2000亿元，达到2178.6亿元。动力电池与电动车、生物与新医药、新型膜材料等新兴产业发展取得突破，建成国内第一家电动车充换电站，新能公司年产50万辆电动车、双鹭生物医药产业园、煤制乙二醇、甲醇制烯烃等项目开工，中原软包装科技园一期工程、华兰生物1000万人份基因重组乙肝疫苗等项目竣工投产。由中国两院院士评出包括绕月工程在内的2010年全国十大发明中，仅有的两项煤化工技术乙二醇和烯烃均落户新乡。成功创建国家火炬计划生物医药特色产业基地、新型电池及材料特色产业基地和长垣起重机械产业基地。高新技术产业增加值比重达到30%，居全省前列。农业基础地位不断巩固。实施优质粮高产开发工程，粮食总产连续5年创历史新高，达到76.2亿斤。粮经比达到60:40，畜牧业产值占农业总产值比重达到38.3%。龙头企业和农民专业合作组织辐射带动农户达到50%。认证地理标志农产品、绿色食品、无公害农产品数量均居全省前列。坚持不懈地推进科技兴农，初步构建了以国家、省、市三级农业科研院所和国内外知名龙头企业为支撑的现代农业发展体系。服务业发展提速。浙江海宁皮革城、杭州四季青服装城、澳门宝龙、菲律宾SM集团、红星美凯龙家居等著名商贸企业入驻我市。市职业技术学院和长垣博大烹饪学校升专成功，全市职教在校生达到11万人。旅游接待人数和综合收入增幅近两年保持在全省前2位。万村千乡市场工程、新网工程、政府早餐示范工程积极推进，家电和汽车、摩托车下乡政策全面落实，消费市场不断扩大。

　　过去五年，项目建设成效显著，发展后劲明显增强。坚持“引资项目双带动”战略，各级主要负责同志全力以赴谋招商、抓招商，对重大项目紧盯不放，直接推动了总投资100亿元的中兵集团通信科技工业园、100亿元的中航集团产业基地、100亿元的中国软包装产业基地、27亿元的百威啤酒基地、20亿元的上海胜华电缆产业园、19.6亿元的北新建材中部建材城等重大项目落地和实施，与20余家央企建立了战略合作关系，提前两年完成“十一五”利用外资目标。建立完善市级领导分包、项目例会等工作推进机制，全力加快重点项目建设步伐。工业投资占城镇投资比重连续四年超过60%，在全省4次重点项目建设观摩中，前3次均居豫北片区第1名。新开工亿元以上工业项目303个，竣工242个，初步扭转了我市缺乏重大工业项目支撑的局面。京港澳高速拓宽工程、宝山电厂2×66万千瓦火电机组、赵固一矿年产240万吨原煤等项目竣工，石武客专、新晋高速、渠东热电2×33万千瓦热电联产等项目开工，新增公路里程8908公里、发电装机容量312万千瓦，交通、能源保障体系进一步健全。

过去五年，统筹城乡发展实现破题，整体形象明显提升。城镇化率达到42.76%，高于全省 3.2个百分点。我市统筹城乡工作的具体实践得到了中央、省及社会各方面的充分肯定，在中央农村工作会议上作了典型发言，成为全省统筹城乡发展试验区。新型农村社区建设积极推进。启动建设327个，完成建筑面积1309万平方米，入住农户5.7万户，52个示范社区功能不断完善。社区户籍管理、社会保障、医疗卫生、教育体制等方面的改革取得初步成效。产业集聚区建设加快。在全省率先实施产业集聚区建设，各级产业集聚区建成区面积由97平方公里增加到136平方公里，规模以上工业总产值占全市的73%。平原新区基础设施建成区面积达到30平方公里，成为全省六大新区之一。城乡建设管理水平提高。深入开展“新城杯”竞赛，积极实施“3010”民生工程行动计划，市区和县城建成区面积由171平方公里增加到243平方公里。市区完成棚户区和旧城改造420万平方米，城中村拆迁143万平方米；8个县（市）近三年完成旧城拆迁500万平方米，建成350万平方米。主城区到“三区五城”8条快速路全部通车。累计投入1700万元，构建了以数字城管为支撑的大城管格局，初步形成了全覆盖、无缝隙、精细化、信息化、长效化城市综合管理体制，市民生活工作环境明显改善。城市形象明显提升。相继获得国家卫生城市、国家园林城市、国家森林城市、全国社会治安综合治理优秀市、全国创建文明城市工作先进市、中国城市综合竞争力百强市、中国最佳管理城市、国家知识产权示范创建城市、中部最佳投资城市等一系列含金量较高的荣誉，连续三届荣获“全国质量兴市先进市”、连续四届荣获“全国双拥模范城”称号，新乡的知名度显著提升。

过去五年，改革创新力度加大，发展活力明显增强。国有企业改制全面完成。坚定不移、锲而不舍地推进国企改制工作，努力克服财力薄弱、安置职工总量大等实际困难，完成268户国有（集体）企业改制任务，妥善安置职工21.2万人，盘活资产227.9亿元，使企业机制得到彻底转换，迸发出了巨大的发展动力与活力。警务机制改革创新模式全省推广。在全省率先推进警务机制改革，实现四警合一、一警多能、综合执法、警力下沉，基层派出所警力占到市区总警力的70％，运行机制逐步健全，服务能力不断提升，公众安全感指数居全省前列。要素保障能力不断增强。整合政府投融资平台，组建新乡投资集团，融资能力达到80亿元；上海万利和欧瑞资本在我市成立创业投资基金；全市银行业金融机构、证券期货经营机构分别达到13家和5家，上市企业3家。共批准建设用地14.86万亩，争取城乡增减挂钩指标1.1万亩，建设用地量名列全省前茅；争取到总投资28.86亿元的南水北调渠首及沿线土地整治重大项目并正式启动；建成标准厂房639万平方米。自主创新走在全省前列。实施省“十一五”重大科技专项11项，占全省的五分之一，培育国家高新技术企业34家。省级以上企业研发中心达到118家，居全省前列。专利申请量连续六年突破千件，研制出全国首例甲型H1N1流感疫苗，攻克了锂离子电池用高分子纳米微孔膜制备等一批关键技术，开发出核苷核酸系列新产品。扶持驻新高校发展，成立本科院校新乡学院，高等院校达到7所，数量居全省第2位。加强产学研结合，80%的规模以上企业与科研院所、高校建立了长期合作关系。科教兴市战略取得新成绩。

过去五年，社会事业全面进步，群众得到较多实惠。努力改善民生，直接用于民生的财政资金达到220.7亿元，占一般预算支出的40.8%，省市承诺的实事全面完成。城镇新增就业44.4万人，下岗失业人员再就业30万人，农村劳动力转移就业542.9万人次，在全省率先提出消除零就业家庭并实施动态管理，城镇登记失业率4.1%。在全国率先实施全区域新型农村合作医疗，参合率达到99.2%，居全省第1位，建成标准化村卫生室1883所；在全省率先实现企业养老保险、失业保险市级统筹；辉县市、新乡县、封丘县被批准为新型农村养老保险试点，覆盖全民的社会保障体系逐步健全。全面普及义务教育，基本普及高中阶段教育，80万中小学生全面享受免费义务教育，“联合校”试点成效明显，进城务工子女入学实现应入尽入、同城同待遇。建成农村文化大院1033个。解决了34092户住房困难群体的住房问题和108万农村人口的饮水安全问题，19.8万农村贫困人口稳定脱贫。人口自然增长率控制在5.3‰以内。强化食品药品监管，加强价格管理调控，对低收入群体发放价格补贴1033万元。广播电视覆盖率达到99%。生态文明建设扎实推进，全市有林地面积达到205万亩，森林覆盖率达到20.9%。各县（市）均建成污水、垃圾集中处理工程。实现了县县通高速、村村通油路。认真解决群众合理诉求，安全生产形势平稳，民族团结不断巩固，应急管理能力逐步增强，连续4年被评为全省平安建设先进市，去年夺取省平安建设最高奖项“中原平安杯”，社会大局和谐稳定。

　　五年来，我们大力弘扬“厚善、崇文、敬业、图强”的城市精神，着力加强精神文明和民主法制建设，深入开展国家文明城市创建活动，城市公共文明指数居全省同类城市第1位，人民群众热爱新乡、奉献新乡的自觉性、主动性不断增强。

五年来，驻新部队积极投身地方建设，在抗旱、抗洪救灾等急难险重任务中发挥了重要作用。五年来，我们着力推进各项事业均衡发展，统计、审计、史志、人防、地质、气象、测绘、地震、社科研究等工作取得新的成绩。五年来，我们不断深化行政管理体制改革，积极转变职能，扎实开展反腐败斗争，政府自身建设取得新的成效。

各位代表：五年来的巨大变化，印证了党中央、国务院和省委、省政府及市委的正确决策和战略部署，记载着历届市领导班子的传承接力和全市人民的团结拼搏，凝聚着社会各界的聪明才智和辛勤汗水，饱含了驻新单位的大力支持和积极帮助。在此，我谨代表市人民政府，向所有参与、支持、关心新乡发展的同志们、朋友们表示衷心的感谢和崇高的敬意！

各位代表：五年来的成功实践，提升了我们的执政理念、发展理念和工作理念，积淀了在复杂环境中推动科学发展的宝贵经验。我们的主要体会是：必须坚持发展这个第一要务。要始终坚持以科学发展观统领经济社会发展全局，牢牢把握发展这个第一要务，凡是认定的目标、看准的事情，都坚定不移、全力以赴地狠抓落实；凡是实践证明正确的思路，都一以贯之、毫不动摇地坚持实施。必须倾听群众的第一呼声。要始终带着对人民群众的深厚感情去思考发展、决策工作、处理问题。凡是关系群众现实利益的各种问题，都在发展的基础上，逐年加大投入，逐步加以解决；凡是事关群众长远利益的工作，都全力以赴，努力克服困难，扎实推进。必须突出创新这个第一动力。要始终坚持用创新的思路、创新的机制、创新的举措来推动工作。凡是上级的决策部署，都要坚决贯彻落实，并结合新乡实际，创造性地加以实施；凡是有利于新乡长远发展的工作，都不等不靠，敢为人先，超前谋划，稳妥推进。必须强化团结这个重要保障。要始终坚持团结协作，汇集各方力量，努力形成合力攻坚、甘于奉献的浓厚氛围。诸多成绩是靠全市上下、方方面面经过艰苦细致、反复做工作取得的；许多重点工作是靠人民群众的理解、支持和参与而顺利推进的。这些都是新乡发展的宝贵财富，必须在今后的工作中加以巩固和发扬。

各位代表：在肯定成绩的同时，我们也清醒地认识到，经济社会发展中还存在不少矛盾和问题。一是晋位升级压力较大。我市经济总量规模偏小，人均指标偏低，还有一些指标在全省靠后，区域竞争日益激烈。固定资产投资增速不理想。二是结构调整任务较重。三次产业发展不够协调，城乡二元结构依然明显，县域发展不平衡，自主创新能力有待提升，经济增长的内生动力依然不足。三是资源环境约束加剧。一些重要领域的体制机制障碍尚未有效破解，市场化运作水平有待提高。资金、土地、资源等瓶颈制约因素较多。环保形势依然严峻，农村环保问题突出，节能减排压力加大。四是民生改善任务艰巨。农业基础地位薄弱，极端天气变化增多，粮食连续增产、农民持续增收难度加大。社会事业发展滞后，公共服务水平不高。当前物价上涨压力较大，群众在上学、就医、住房等方面还不尽满意。五是自身建设有待加强。部分单位依法行政观念不强，办事效率不高。一些干部和工作人员服务意识淡薄，作风不够扎实。极少数干部以权谋私、腐化堕落。我们将在今后的工作中着力解决这些问题。

　　二、“十二五”规划概要

随着全球经济一体化进程加快，调整转型将成为今后五年最重要的阶段性特征；科技创新孕育新突破，以新兴产业为重点的经济再造时代全面到来。经历国际金融危机之后，我市发展逆势上扬，主要经济指标晋位升级，重点工作不断突破，进入了新一轮上升周期。未来五年是新乡跨越赶超的黄金机遇期。我们要准确把握形势变化和阶段性特征，抢抓机遇，争创优势，再创辉煌。

“十二五”时期我市经济社会发展的总体思路是：以科学发展为主题，以转变经济发展方式为主线，以富民强市为中心任务，紧紧围绕项目建设，着力打造中原经济区统筹城乡发展示范区、先进制造业基地、现代农业试验示范基地和高素质人力资源培育基地等“一区三基地”，更加注重民生改善，保持社会和谐稳定，实现综合实力迈入全省前列，建设中原经济区强市，提升人民的幸福指数。

要实现综合实力迈入全省前列，在指导思想上，必须以科学发展为总原则，突出三个发展：一是转型发展。把加快转变经济发展方式贯穿于经济社会发展全过程和各领域，着力推进领导方式、产业结构和经济增长方式转变。正确处理发展与转变的关系，坚持在发展中转变、在发展中调整、在发展中提升、在发展中增效。二是跨越发展。实现地区生产总值年均增长12%左右，城镇化率高于中部地区平均水平，保持跨越发展的态势，努力在体现现代产业体系水平、富民强市的主要经济社会指标、城乡建设面貌、人民群众的幸福感指数等四个方面走在全省前列。三是和谐发展。坚持富民优先，协调发展，在社会建设、民生改善、环境保护等方面全面进步，构建和谐社会新局面。

要实现综合实力迈入全省前列，在工作推进上，必须大力培育新的经济增长极和增长点，强化发展支撑：一是产业支撑。以生物与新医药、电池电动车与汽车零部件、电子信息、特色装备制造、制冷、现代煤化工等六大主导产业为支撑，培育壮大新兴产业，改造提升食品、纺织、造纸、建材、能源等传统优势产业，用三到五年时间，再造一个全新的工业体系。二是项目支撑。强力实施引资项目双带动战略，再引进一批投资额度大、科技含量高、带动作用强的基地型项目，充分发挥重大项目的支撑带动作用。三是区域支撑。加快中心城区、平原新区、县城、产业集聚区和重点镇建设步伐，培育一批新的经济增长板块。四是科技人才支撑。强化产学研结合，引进和掌握一批关键核心技术，争创一批名牌产品，推动高新技术产业发展。加快人力资源开发和引进，打造全国重要的职业教育品牌。五是统筹城乡支撑。把新型农村社区建设作为推进城乡一体化的切入点、统筹城乡发展的结合点和促进农村发展的增长点，推动农村向城镇靠近、城镇向农村延伸，走“三化”协调发展、科学发展之路。六是基础设施支撑。抓好基本农田保护和水利设施建设，确保粮食高产丰收。推进客运铁路、货运铁路、公路路网及电力、煤炭、新能源开发利用等综合交通运输体系和能源体系建设。抓好节能减排和环境保护。七是体制机制支撑。善于发现、总结推广新经验，虚心学习借鉴先进地区好办法，解放思想、勇于创新，有效运作、破解难题。

各位代表：虽然目前我市经济规模仍处于全省中游，但总体结构较好、发展后劲较足、厚积薄发之势已经显现，我们有信心在现有的基础上乘势而上、实现跨越，圆满完成“十二五”的目标任务。《新乡市国民经济和社会发展第十二个五年规划纲要（草案）》及说明已印发各位代表，请一并予以审议。

三、2011年工作安排

今后一个时期，我们既面临产业转移步伐加快、内需拉动不断增强、后发优势日益显现及中原经济区上升到国家层面等难得机遇，也存在结构性矛盾突出、资源环境约束加剧、区域竞争压力加大、改善民生任务繁重等诸多困难。总体判断，今年发展面临的有利条件增多，机遇大于挑战。我们要坚定信心、抢抓机遇，开创跨越发展的新局面。

今年经济社会发展主要预期目标是：生产总值增长12.5%，其中一、二、三次产业分别增长3.8%、15.5%和10.5%。全社会固定资产投资增长19%，其中城镇投资增长20%，工业投资增长15%以上。社会消费品零售总额增长16%。地方财政一般预算收入增长14%。城镇居民人均可支配收入增长10%，农民人均纯收入增长10%以上。城镇新增就业7万人，城镇登记失业率控制在4.5%以内。人口自然增长率控制在6.5‰以内。节能减排指标控制在省定目标之内。重点抓好九个方面的工作：

　　（一）提升招商引资工作水平。要有超常规的思路和措施。围绕“人、政策、环境”三个核心要素，配强招商队伍，重奖引资功臣。各县（市）、区要保证一名党政主要负责同志常年在外招商，开展小分队招商50次以上；至少引进1个投资10亿元以上的重大产业项目；引进1个以上外贸出口型企业。对能弥补产业链缺项、带动作用强的基地型项目，不求所有，但求所在，敢于拿出优质资源与国内外大型企业集团嫁接重组。积极承接沿海产业转移。各县（市）、区和产业集聚区要根据自身优势，选定产业、明确目标，改进招商方式，组建专门班子到产业集中、特色鲜明的地区蹲点、驻地招商，吸引沿海产业集群式转移，各县（市）、区至少引进1个产业集群项目。抓好海宁皮革城、杭州四季青服装供应商产业转移项目，加快封丘服装产业园和延津食品工业园建设，争取福建、台湾服装、鞋帽和晋江食品产业集群落地。做好优势产业和龙头企业招商。围绕动力电池与电动车、生物与新医药、现代煤化工、特色装备制造、现代农业等领域，紧盯富士康、中国医药、中国高新、中海油新能源、中油昆仑信托、中国电科、中化集团、中国黄金、中国通用技术、天汽集团、新华联、吉祥建材等企业，争取一批项目落地。下大力气优化环境。分管市领导对重大项目、基地型项目亲自挂帅，组建专门班子，主动搞好服务。坚决兑现对投资人的承诺，严肃查处影响项目落地建设的人和事。全年实际利用外资4亿美元，引进市外内资244亿元，引资总量保持全省前列。抓好口岸作业区建设，完成出口8.2亿美元。

（二）加大重点项目建设力度。抓好重大项目谋划。依托“一谷五基地”、新兴产业、产业集群等载体，在工业、高新技术、基础设施、品牌农业等领域谋划储备一批投资超10亿元、50亿元乃至上百亿元的基地型项目。重点抓好电池与电动车、煤化工下游产业及中科院煤化工实验基地、华兰与双鹭生物医药产业园、豫新2×60万千瓦热电机组、赵固综合利用电厂2×30万千瓦机组、郑新城际铁路等项目的前期工作。积极争取上级政策资金支持，确保省重点项目、省核备市重点项目个数和争取省以上各类资金总量均进入全省前3名。加大投资强度。实施千万元以上项目1670个，完成投资1113亿元，其中亿元以上项目508个，完成投资561亿元。加大中兵通信科技工业园、中航高科技产业园等50个重大工业项目投资力度，确保渠东热电一期工程、中粮（新乡）现代粮食产业化基地、新太行电源产业园、拓新生化生物医药产业园等40个亿元以上项目竣工投产。中国电波科技城、新中益2×60万千瓦火电机组、银金达新型包装膜、济东高速东明黄河大桥等35个亿元以上项目全面开工。形成工作合力。健全重大项目建设工作推进机制，提高重大项目前期手续审批、备案效率。对于不按合同办事、严重影响工程进度的建设承包单位，坚决清除出新乡建筑市场，严厉打击各种违法阻工行为，确保重大项目建设顺利推进。

（三）加快工业转型升级。新乡能否实现跨越发展，新兴产业、优势支柱产业的突破至关重要。要全力加大生物与新医药、动力电池与电动车、电子信息、现代煤化工、新型膜材料等新兴产业和特色装备制造、制冷、汽车零部件等优势支柱产业有关重大项目的实施力度，推动企业重组，培育产业集群，走新型工业化发展道路。生物与新医药：做好中原经济区生物产业园发展规划，支持华兰生物整合国内血液制品企业，发展新型疫苗制剂，打造全国血液制品和新型疫苗产业基地。加快双鹭生物医药基地建设步伐，大力发展基因工程药物。与国药控股合作，打造中国卫生材料产业基地。动力电池与电动车：争取在正负极材料、隔膜、电池质量等方面取得更大突破，加大中科公司锂电隔膜、科隆集团新能源材料产业园区、科隆集团新能源汽车电源动力系统总成、环宇集团1万辆车用动力电池、锂动电源6亿安时动力电池等项目实施力度。发挥材料优势，引进一批最先进的电池、操作系统总成项目和自动生产线、专业实验室等落户新乡，积极吸引有实力的战略投资者和高端人才加盟。加快新能电动车项目建设步伐，争取金龙集团30万辆电动轿车项目尽快开工，确保上半年60辆电动车投入示范运营，建设竞争优势明显的动力电池基地和国内重要的电动车生产基地。积极发挥汽车零部件产业优势，做大做强新飞、高远专用车和新航、万向、欧玛汽配等骨干企业。现代煤化工：做好煤炭和煤层气资源勘探开采工作，抓好中科化工年产60万吨烯烃、中新化工年产20万吨乙二醇、心连心化肥年产80万吨尿素、金天化工年产100万吨联碱等项目建设，千方百计拉长产业链条，加快现代煤化工基地建设步伐。积极谋划、引进和建设煤化工下游产业链项目，大力发展聚合物和新型膜材料，抓好中原软包装科技园二期等项目实施，打造印刷包装产业园。电子信息：发展卫星通信、电波研究成果应用、战术数据链、微波着陆系统、超宽带自组网通信电台等高端产品，加快推进中国电波科技城等项目建设，打造中国信息产业研发生产基地。特色装备制造：加快实施太行振动60台特大振动输送机产业化、科隆集团年产10万吨高效节能换热器等大型煤化装备、豫飞重工新型工业化特色装备产业园等项目，打造全国重要的起重、振动、石化装备和环保装备等特色产业基地。制冷：完善发展规划，出台支持政策，紧盯产业链缺项，引进制冷终端企业。扶持新飞、中航等企业快速发展，加大海宝电器100万台冷柜、展示柜、保鲜柜生产线和科隆集团无氟高效蒸发器（冷凝器）扩产改造等项目的实施力度，打造全国重要的制冷产业基地。改造提升食品、纺织、造纸、建材、能源等传统优势产业，提高产业发展整体水平。引导央企和国内外行业龙头企业参与我市产业重组，争取在新乡扩大规模，建设区域总部、研发中心、营销中心和生产基地。推动市规模效益50强企业和30户高成长性企业快速发展。加强专业化分工协作，鼓励龙头企业专做研发、整机组装和销售，中小企业专搞配套，走产业集群发展道路。

　　（四）大力发展现代农业。全面推进农业产业化、市场化、标准化和组织化，促进农业增效、农民增收，把新乡农业做成全国亮点。稳定粮食生产。抓住国家支持水利建设的大好机遇，加快农田水利基本建设，争取实施引黄调蓄工程项目。组织好中科院的中低产田改造示范项目、国土部的200万亩土地整治项目和国家千亿斤项目。开展小麦、玉米、水稻等大宗农作物高产创建活动，打造河南省粮食生产核心区优质高产示范区。建设优质小麦产业园和中国优质小麦种子基地。粮食总产达到80亿斤以上。优化农业结构。加快中粮一期仓储物流项目建设，确保二期60万吨面粉和30万吨饲料项目落地，打造国家级粮油食品产业园。大力发展现代畜牧业，积极推进粮、畜产品深加工。积极发展林果、蔬菜、苗木花卉、中药材、有机食品、度假休闲等生态高效农业，经济作物总产达到410万吨以上。打造品牌农业。建立科研、生产与流通相结合的市场机制，加快农业科技成果推广和产业化步伐。加强对品牌农产品的培育和包装推广，提高品牌知名度。推动种子企业的重组和跨地域整合，加强与中国种子集团、太空育种中心和中粮集团的战略合作，打造种业品牌。创新农业经营机制。加快农业产业化进程，扶持中粮集团、雨润集团、高金食品、克明面业、五得利面粉、迪一米业、新良粮油、亚特兰食品等龙头企业发展壮大。培育农民专业合作组织，发展专业合作社100家，订单农业面积达到230万亩。按照“依法、自愿、有偿”的原则，推进土地流转，促进规模经营。

（五）提升服务业层次和水平。支持海宁皮革城、杭州四季青服装城及南环物流园、北环物流园、华亚钢材物流园做大做强。科学布局市区商业中心、特色商业街和社区商业服务网点。加快南太行旅游重点项目建设，推进资源整合，实施“百村万户”旅游富民工程，旅游总收入、接待游客人数均增长15%以上。扶持比干诞辰纪念、长垣饮食、辉县百泉药交会等特色文化区块发展。发挥文投公司作用，积极包装项目，加大招商引资力度，培育龙头企业，不断提升文化产业的整体规模和实力。建立“扫黄打非”长效机制，促进文化产业健康发展。大力发展职业教育，推进市卫校、幼师职业教育园区建设，开展农民工职业技能培训4万人次，培养中高级技师1000人。抓好万村千乡市场工程和新农村现代流通服务网络工程建设，完善放心早餐工程服务体系。落实好家电和摩托车下乡政策，扩大农村消费。

（六）深入推进统筹城乡发展。争取并启动实施全国农村改革试验区工作，破解资金、土地难题，努力探索更多经验。推进新型农村社区建设。坚持“规划先行、就业为本、群众自愿、量力而行、因地制宜、分类推进”的原则，以52个示范社区建设为重点，完成投资65亿元，新增建房800万平方米，新入住农户4万户；拆除旧宅1.8万亩，复耕或恢复生态1万亩。积极探索城乡一体化制度建设。加快产业集聚区建设。全面加快基础设施建设和项目引进，各级产业集聚区完成基础设施投资130亿元，每个省级产业集聚区开工建设10个亿元以上项目，每个专业园区开工建设10个5000万元以上项目。进一步理顺体制，完善机制，确保省级产业集聚区在现有排名基础上只升不降，高新技术产业开发区成功创建国家级高新技术开发区，新乡工业园区争创国家级经济技术开发区。抓好平原新区建设，完成固定资产投资35亿元以上，引进电动车、生物与新医药、电子信息等新兴产业。加强城市建设和管理。实施城乡建设三年大提升行动计划，突出抓好新东区石武高铁车站广场及周边基础设施建设，全面推进107国道改造和景观建设。加快新晋高速、鹤辉高速、南太行旅游环线道路等重点项目建设，开工S227西曹线延津至封丘段改建工程。实施“公交优先发展”战略，增加一定数量的公交车，启动市区环城“BRT”建设。加快市区水源地建设，改造超期服役和存在隐患的各类管网，治理交通堵塞，强化高层楼房消防安全管理，有效防止“城市病”。市区完成棚户区和城中村拆迁300万平方米以上。加快直升机场搬迁，以此为契机，加大城市西区开发建设力度。谋划实施凤泉湖、新乡医疗健康园区等项目建设和市殡仪馆搬迁工作。强化资质审查，完善招标条件，理顺建筑市场管理体制。推动“数字城管”向下延伸，提升县城和重点镇建设管理水平。城镇化率提高1.8个百分点以上。

（七）强化改革创新。加大市场化运作力度。全面推进基础设施建设市场化融资。新兴产业要积极引进战略投资、风险投资基金和各类合作伙伴。财政扶持引导资金要多采用投资入股办法。加强银企对接，新增贷款80亿元以上。做大投资集团，新增融资15亿元以上；支持新乡银行引进战略投资者。扶持中小企业担保集团发展壮大，加快中小企业集合票据发行工作。争取金龙铜管、日升数控年内上市，申报一批上市企业。抓好乡村规划和村庄合并，加快土地流转和置换，筹措建设资金。深化各项改革。做好市直部门所属企业脱钩改制工作，健全国有资产监管体系。合理调整区划，促进区域均衡发展。做好事业单位分类改革。推进县（市）公安机关警务机制改革创新。深化医药卫生体制改革，全面实施国家基本药物制度，完善医药配送领域的竞争、择优机制，减轻群众就医负担。探索文艺院团转企改制新道路。构筑区域自主创新体系。实施动力电池材料研发与产业化等3项省级重大科技专项，新增省级以上研发中心2家。确保国家电池质检中心挂牌、河南省振动机械产品检测站投入运行。新增省级以上名牌产品10个，专利申请量继续保持在1000件以上。积极引进各类高层次专业人才，支持新兴产业相关企业引进高级人才，扶持驻新高校发展，加强产学研结合，打造高素质人力资源培育基地。

　　（八）大力保障和改善民生。加大民生和社会事业投入，强化财政资金使用监管，全面完成省市承诺的实事办理工作。千方百计增加就业。加快创业孵化园建设，发放小额担保贷款3.1亿元，下岗失业人员再就业2.4万人，就业困难对象再就业0.9万人，应届高校毕业生就业率达到80%以上，确保国家级创业型城市通过验收。健全社会保障体系。新增城镇基本养老保险参保人数2万人。解决5000名未参加养老保险的集体企业、“五·七工”、“家属工”、返乡老知青等群体的参保问题。再争取2个全国新型农村社会养老保险试点县，新型农村社会养老保险参保人数达到140万。将城镇居民医疗保险补助金由120元提高到200元。新型农村合作医疗补偿封顶线由6万元提高到10万元。提高农村最低生活保障和农村五保供养标准，五保集中供养标准由每人每年不低于2000元提高到2240元，分散供养标准由每人每年1200元提高到1320元。落实国家房地产调控政策，增加有效供给，抑制投机需求，引导合理消费。建成保障性住房13658套、96万平方米。解决好农民工在劳动报酬、子女就学、住房租赁、社会保障等方面的实际问题。大力发展教育事业。加大教育投入，每个县（市）、区至少建1所公办直属幼儿园。每个乡镇建设1所寄宿制初中，切实解决农村留守儿童的上学问题。扩大“联合校”实施范围，缓解择校压力。编制城镇中小学布局专项规划，促进义务教育均衡发展。加快中小学校舍安全工程建设，完成投资2.7亿元、改造校舍28万平方米。加快医疗卫生事业发展。开工建设市健康科技园区、市中心医院门诊楼、市全科医师培训基地；启动市妇幼保健院搬迁项目、中心医院与北京双鹭合作建设新区医院项目；推进市结防所搬迁。建成区域卫生信息平台，实现参合农民就医一卡通和即时结报。新建500所标准化村卫生室。为3800名贫困白内障患者免费实施复明手术。推行临床路径管理、按病种付费，控制药占比。做好重大传染病防控。加强物价监测和调控。抓好“米袋子”和“菜篮子”工程，加大价格监管和价格执法力度，完善补贴制度和储备制度，落实应急预案，维护市场稳定。加强食品药品监管，确保食品药品安全。强化节能减排和环境保护。抓好国家环保模范城创建，建成孟庄、小店、平原新区、贾屯污水处理厂并确保稳定运行。全面实施水环境生态补偿，严厉打击环境违法行为，确保出境断面水质、饮用水水源地水质达标。加大大气污染整治力度，城市环境空气质量优良天数达到85%以上。加强农村环境保护，控制面源污染。强力推进林业生态市建设，完成林业生态建设任务15万亩。加大“全国绿化模范城市”创建力度，确保各项指标达到创建标准。抓好结构减排、工程减排和监管减排。淘汰落后产能、工艺和技术设备，积极调整高耗能行业产品结构，建立健全项目节能评估和审查制度，坚决杜绝“两高一资”项目。促进社会事业全面发展。改造危桥15座，保障群众出行需要。再解决20万农村居民安全饮水问题。新建、改建农村公路500公里。实现4.5万农村贫困人口脱贫。新建农家书屋838个。全力推进以TD和3G网络为依托的无线城市建设。加强社区平台建设，健全老龄组织，开展居家养老试点。稳定低生育水平，维护人口结构安全。积极开展法律援助，依法保护妇女、未成年人、残疾人和老龄人的合法权益。抓好未成年人思想道德建设。加强城乡体育设施建设，启动平原体育（会展）中心、老体育中心建设与改造，广泛开展全民健身运动。做好民族宗教工作，深入开展民族团结进步先进市创建活动。大力支持驻新部队建设，持续开展双拥共建，巩固军政军民团结。争创全国文明城市，推进国家健康城市建设。加强社会治安综合治理，抓好“安全新乡”创建，有效防止重特大事故发生。加强和创新社会管理，做好基层基础工作，建立完善调处化解矛盾纠纷平台，解决群众合理诉求，提高妥善处理群体性事件、突发事件和网络媒体报道焦点的能力和水平。逐步增加灾害救助投入，提高自然灾害应对能力。健全预防预警和应急处置体系，维护社会和谐稳定。充分发挥工会、共青团、妇联、文联、侨联、社科联、残联等人民团体的桥梁纽带作用。做好新闻出版、广播电视、防震减灾、统计、气象、史志、外事、侨务、档案、测绘、人防等工作，促进各项社会事业均衡发展。

　　（九）加强政府自身建设。建设人民满意政府是一项持久而紧迫的任务。认真落实“四个重在”实践要领，按照“三具两基一抓手”工作方法，深入开展“两转两提”活动，进一步提高领导和推动科学发展的能力。着力民生民心。努力改善民生，高度关注困难群体的疾苦，认真落实惠民富民的政策措施，多做雪中送炭的事，坚决纠正损害群众利益的不正之风。转变领导方式。解放思想，勇于创新，努力在项目建设、招商、融资、统筹城乡、发展战略新兴产业等方面有效运作，加快发展。精简会议、文件，清理和规范各种达标评比活动，注重实干实效，狠抓工作落实。坚持依法行政。自觉接受人大及其常委会的法律监督、工作监督和政协的民主监督，广泛接受社会公众和新闻舆论监督。健全重大决策专家论证、技术咨询、风险评估以及听证、公示等制度，促进决策科学化、民主化、规范化。转变政府职能。强化社会管理和公共服务职能，推进政企、政事、政资、政府与中介组织分开，深化行政审批制度改革，不断提高服务人民、服务发展的能力和效率。自觉廉洁从政。认真落实党风廉政建设责任制，健全权力运行监督制约机制，加大腐败案件查处力度。坚持公开、公平、公正和竞争、择优原则，加快 “一费制”改革步伐，强化公共资源交易中心、行政服务中心建设，规范土地出让、资源开发、项目招标、行政审批、政府采购、资金安排、国有资产转让、重大工程建设等管理，探索从源头上、机制上防止腐败的有效措施。坚持勤俭节约、勤俭办事，年内公务购车、出国、接待经费要有明显下降，确保年度一般预算支出零增长，坚决反对奢侈浪费。

各位代表!站在历史的新起点，面对人民的新期待，我们深感责任重大、使命光荣。让我们紧密团结在以胡锦涛同志为总书记的党中央周围，高举中国特色社会主义伟大旗帜，深入贯彻落实科学发展观，奋发图强，扎实工作，谱写新乡跨越发展的新篇章！
